# Diagnostic value of combined CT artificial intelligence (AI) system and lung cancer biomarkers in pulmonary nodule evaluation

**DOI:** 10.3934/publichealth.2025059

**Published:** 2025-12-15

**Authors:** Lile Wang, Shuying You, Jianyi Zhou, Mo Liang, Ruicheng Hu

**Affiliations:** 1 Department of Respiratory Medicine, Hunan Provincial People's Hospital, The First Affiliated Hospital of Hunan Normal University, Changsha 410016, China; 2 Department of Respiratory Medicine, The Second People's Hospital of Hunan Province, Brain Hospital of Hunan Province, Changsha 412007, China

**Keywords:** CT/AI system, lung cancer biomarkers, pulmonary nodules, sensitivity, specificity

## Abstract

**Objective:**

To analyze the diagnostic value of a Computed Tomography (CT) artificial intelligence (AI) system combined with lung cancer biomarkers for pulmonary nodules.

**Methods:**

A retrospective analysis was conducted on 200 patients with pulmonary nodules treated at our hospital from February 2021 to January 2025. Based on pathological results, patients were divided into a benign group and a malignant group. The two groups were compared in terms of baseline data and lung cancer biomarkers, including carcinoembryonic antigen (CEA), neuron-specific enolase (NSE), cytokeratin 19 fragment 21–1 (CYFRA 21–1), squamous cell carcinoma antigen (SCCA), and pro-gastrin-releasing peptide (ProGRP). The sensitivity, specificity, accuracy, misdiagnosis rate, and missed diagnosis rate of the CT/AI system alone and in combination with lung cancer biomarkers were analyzed.

**Results:**

There were no statistically significant differences between the benign group (134 cases) and malignant group (66 cases) regarding sex, lobulation sign, spiculation sign, solitary pulmonary nodule (SPN), or mean CT value (P > 0.05). However, the benign group had significantly lower age, years of smoking, chronic lung disease, pure ground-glass nodules (pGGN), nodule diameter, irregular nodules, bronchial changes, and vascular changes compared to the malignant group (P < 0.05). Levels of CEA, NSE, CYFRA 21–1, SCCA, and ProGRP were also significantly lower in the benign group than in the malignant group (P < 0.05). Taking pathology as the reference standard, the CT/AI system alone had a sensitivity of 71.21% (47/66), specificity of 85.07% (114/134), accuracy of 80.50% (161/200), misdiagnosis rate of 19.50% (39/200), and missed diagnosis rate of 28.79% (19/66). In contrast, the CT/AI system combined with lung cancer biomarkers had a sensitivity of 92.42% (61/66), specificity of 93.28% (125/134), accuracy of 93.00% (186/200), misdiagnosis rate of 7.00% (14/200), and missed diagnosis rate of 7.58% (5/66), with all diagnostic parameters significantly improved compared with the CT/AI system alone (P < 0.05). Logistic regression analysis showed that age, smoking for >20 years, chronic lung disease, nodule diameter, irregular nodules, bronchial changes, vascular changes, NSE, CYFRA 21–1, and SCCA were all risk factors for malignant pulmonary nodules (P < 0.05). Receiver operating characteristic (ROC) curve analysis demonstrated that age, nodule type, chronic lung disease, nodule morphology, bronchial changes, and vascular changes had modest value for predicting malignant pulmonary nodules, with AUCs of 0.586, 0.750, 0.707, 0.601, 0.580, and 0.565, respectively. Smoking, nodule diameter, CEA, NSE, CYFRA 21–1, SCCA, and ProGRP had better predictive value, with AUCs of 0.840, 0.944, 0.958, 0.922, 0.856, 0.978, and 0.990, respectively. The combined diagnosis of all indicators achieved an AUC of 0.993.

**Conclusion:**

The CT/AI system combined with lung cancer biomarkers demonstrates high sensitivity and specificity in diagnosing the nature of pulmonary nodules. Moreover, the occurrence of malignant pulmonary nodules is significantly associated with factors such as age, smoking, and chronic lung disease.

## 1. Introduction

Pulmonary nodules are solid or subsolid localized areas of increased density within the lung that are detected during chest X-ray or CT examinations. They can be caused by various factors, including pulmonary inflammation, granulomatous diseases, and abnormal cellular proliferation [1]. Among them, benign nodules account for approximately 80%–90% of all detected pulmonary nodules, while the detection rate of malignant nodules is closely related to the duration of smoking and increasing age [2],[3]. Vindum et al. [4] conducted a retrospective cohort study involving 4181 patients with pulmonary nodules and found that only 6% of pulmonary nodules developed into lung cancer over a three-year CT follow-up period, and 77.9% of lung cancer patients were able to receive curative treatment. This suggests that timely diagnosis through CT in the early or precancerous stages of lung cancer can effectively improve early prognosis and help control disease progression. Artificial intelligence (AI) can analyze, summarize, and model massive amounts of data using different algorithms and behavior simulations to intelligently integrate the pathological characteristics and biological manifestations of specific diseases. This enables AI to be widely applied in many medical fields, including oncology and radiological imaging [5].

However, the early onset of lung cancer is characterized by concealment and complexity. When the lesion is less than 3 cm in diameter, its impact on lung function is not obvious, and relying solely on a CT/AI system to determine the nature of pulmonary nodules may still result in missed or incorrect diagnoses, thereby affecting the prevention and treatment of precancerous lesions or early-stage lung cancer [6]. Carcinoembryonic antigen (CEA) is a common tumor marker, and a meta-analysis of non-small cell lung cancer (NSCLC) [7] showed that CEA is significantly associated with epidermal growth factor receptor (EGFR) mutations and lung cancer staging. Neuron-specific enolase (NSE) is a glycolytic enzyme primarily distributed in neuronal cells that has high diagnostic and predictive value for small cell lung cancer (SCLC) and tumors of the nervous system [8]. Additionally, NSE, together with other tumor markers such as cytokeratin 19 fragment 21–1 (CYFRA21–1) and squamous cell carcinoma antigen (SCCA), can be used in the diagnosis of different types of lung cancer [9]. However, it has been suggested [10] that the sensitivity and specificity of NSE are inferior to those of pro-gastrin-releasing peptide (ProGRP), indicating that its diagnostic value for pulmonary nodules or lung cancer remains limited. Based on this, the present study analyzes the value of combining a CT/AI system with lung cancer biomarkers in diagnosing pulmonary nodules, with the aim of improving the diagnostic accuracy of malignant pulmonary nodules and early-stage lung cancer, thereby optimizing clinical outcomes.

## 2. Subjects and methods

### 2.1. Study subjects

A total of 200 patients with pulmonary nodules treated at our hospital from February 2021 to January 2025 were selected for retrospective analysis. All study data were collected during treatment or follow-up periods. Among them, there were 124 males and 76 females, aged 35–82 years, with a mean age of 57.55 ± 9.83 years.

(1) Inclusion criteria: ① Patients aged ≥18 years who provided signed informed consent and whose participation was approved by the hospital ethics committee. ② Presence of pulmonary nodules confirmed by chest CT or X-ray examination. ③ Use of AI-assisted systems during the diagnostic process for pulmonary nodules. ④ Availability of pathological and lung cancer-related examination results, such as sputum cytology, percutaneous lung biopsy, and tumor markers for lung cancer. ⑤ Newly diagnosed patients without a history of cardiothoracic surgery, radiotherapy, chemotherapy, immunotherapy, or targeted therapy.

(2) Exclusion criteria: ① Presence of malignancies in other sites, congenital immunodeficiency diseases, or hematological disorders. ② Combined with acute cerebral infarction (ACI), craniocerebral trauma, sepsis-associated encephalopathy, Alzheimer's disease (AD), epilepsy, or other neurologically impairing conditions. ③ Severe anemia, malnutrition, or active gastrointestinal bleeding, such as gastric or duodenal ulcer or erosive esophagitis. ④ Presence of infectious diseases, severe cardiovascular or cerebrovascular diseases, hemodynamic instability, or severe hepatic or renal dysfunction. ⑤ Incomplete clinical, imaging, or pathological data, or women who were pregnant or breastfeeding. ⑥ Patients with implanted stents or metallic devices in the body.

### 2.2. Examination methods

#### 2.2.1. CT/AI examination

All patients underwent examination using the NeuViz 128 In-Mobile multi-slice spiral CT system (Neusoft Medical Systems Co., Ltd.). Before the scan, patients were instructed to remove any metal objects from their body and lie supine on the CT table. Under the guidance of the technician, patients were asked to take a deep breath and hold it to ensure full lung expansion. After positioning, images were obtained, and plain scanning was performed from the lower neck to the diaphragm, covering a range of approximately 35–40 cm. The scanning parameters were as follows: tube voltage 120 kV, tube current 220–300 mA, pitch 0.5–1.0 mm, slice thickness 1.0 mm, reconstruction interval 1.0 mm. The lung window was set to a width of 1700 HU and a level of –650 HU, and the mediastinal window to a width of 320 HU and a level of 35 HU. Following plain scanning, 50–100 mL of iodine-based contrast agent could be administered as appropriate, at a flow rate of 3 mL/s, for contrast-enhanced scanning of the nodule. After the scan, imaging data were imported into the AI-assisted diagnostic system (Shukun Technology Co., Ltd.). Using a high-spatial-resolution sharp algorithm, the system intelligently analyzed features such as nodule vascular supply and vessel traversal, size, type, edge characteristics, and average CT value. These results were combined with radiologists' image interpretations and lung cancer biomarker levels to determine the nature of the pulmonary nodules.

#### 2.2.2. Grouping criteria

Risk stratification of pulmonary nodules was performed according to the Chinese Expert Consensus on the Diagnosis of Early Lung Cancer (2023 Edition) and the 2015 British Thoracic Society (BTS) guidelines [11],[12].

According to the Brock model, nodules with a malignancy probability <10% (solid or subsolid) were defined as low risk, and annual CT follow-up was recommended; nodules with a malignancy probability of 10%–70% were defined as intermediate risk, requiring enhanced CT or tissue biopsy for qualitative assessment, followed by CT review every 3–6 months; nodules with a malignancy probability >70% were defined as high risk, for which histological diagnosis was strongly recommended, followed by appropriate surgical, radiotherapeutic, or chemotherapeutic management based on the results.

#### 2.2.3. Detection of lung cancer biomarkers

Five milliliters of venous blood were drawn from each patient and centrifuged at 3000 rpm for 10 minutes (centrifuge radius 10 cm). After standing, the supernatant was collected. Carcinoembryonic antigen (CEA), neuron-specific enolase (NSE), and cytokeratin 19 fragment 21–1 (CYFRA21–1) were measured using chemiluminescence immunoassay, while squamous cell carcinoma antigen (SCCA) and pro-gastrin-releasing peptide (ProGRP) were measured using enzyme-linked immunosorbent assay (ELISA). All reagents were purchased from Shenzhen New Industries Biomedical Engineering Co., Ltd., and concentrations were uniformly measured using the Stream SuperB-800 fully automated biochemical analyzer (Guangzhou DaAn Gene Co., Ltd.). Reference ranges for each marker were based on the recommended values provided by the assay methods.

### 2.3. Observation indicators

Basic patient data were collected, including sex, age, smoking history, chronic lung disease, nodule type, nodule diameter, and nodule morphology. Referring to the Chinese Expert Consensus on the Diagnosis of Early Lung Cancer (2023 Edition) [11] and the Chinese Expert Consensus on Diagnosis and Treatment of Pulmonary Nodules (2024 Edition) [13], and using pathological results as the gold standard, the sensitivity, specificity, accuracy, misdiagnosis rate, and missed diagnosis rate of the CT/AI system alone and combined with lung cancer biomarkers in diagnosing pulmonary nodules were analyzed. Sensitivity was calculated as true positives / (true positives + false negatives) × 100%; specificity as true negatives / (true negatives + false positives) × 100%; accuracy as (true positives + true negatives) / total number × 100%; misdiagnosis rate as (false negatives + false positives) / total number × 100%; and missed diagnosis rate as false negatives / confirmed cases × 100%.

### 2.4. Statistical analysis

Data were corrected and processed using SPSS version 27.0 software. Count data were recorded as n (%), and comparisons between two independent sample rates or composition ratios were analyzed using the chi-square test. Multiple group comparisons were performed using the z-test. Measurement data were expressed as mean ± standard deviation (±SD) and analyzed with the grouped t-test. Multivariate logistic regression analysis was used to identify risk factors for malignant pulmonary nodules. The predictive value of various indicators for malignant pulmonary nodules was assessed using the area under the receiver operating characteristic (ROC) curve (AUC). A p-value < 0.05 was considered statistically significant.

## 3. Results

### 3.1. Analysis of diagnostic performance of the CT/AI system combined with lung cancer biomarkers

Using pathological results as the reference standard, among the 200 patients, 66 had malignant nodules and 134 had benign nodules. The CT/AI system detected 67 cases of malignant pulmonary nodules, while the CT/AI system combined with lung cancer biomarkers detected 70 cases. The diagnostic sensitivity, specificity, accuracy, misdiagnosis rate, and missed diagnosis rate of the combined method were all significantly better than those of the CT/AI system alone (P < 0.05), as shown in Tables 1 and 2.

**Table 1.** Comparison of diagnostic performance of the CT/AI system combined with lung cancer biomarkers (n).

Pathologic diagnosis	CT/AI		CT/AI + Lung cancer biomarkers	
	malignant	benign	malignant	benign
Malignant (n = 66)	47	19	61	5
Benign (n = 134)	20	114	9	125

**Table 2.** Diagnostic performance analysis of the CT/AI system combined with lung cancer biomarkers (%).

Diagnostic method	Sensitivity	Specificity	Accuracy rate	Misdiagnosis rate	Miss rate
CT/AI	71.21	85.07	80.50	19.50	28.79
CT/AI + Lung Cancer biomarkers	92.42	93.28	93.00	7.00	7.58
*x^2^*	9.982	4.679	13.594	13.594	9.982
P	0.002	0.031	<0.001	<0.001	0.002

### 3.2. Comparison of baseline data

There were no statistically significant differences between the two groups in terms of sex, lobulation sign, spiculation sign, solitary pulmonary nodule (SPN), or average CT value (P > 0.05). However, the benign group showed significantly lower values in age, years of smoking, chronic lung disease, pure ground-glass nodules (pGGN), nodule diameter, irregular nodules, bronchial changes, and vascular changes compared to the malignant group, with all differences reaching statistical significance (P < 0.05), as shown in Table 3.

**Table 3.** Comparison of baseline data between the two groups (*x* ± s, n).

Item		Benign group (n = 134)	Malignant group (n = 66)	*x^2^/t/z*	*P*
Sex	Male	84	40	0.081	0.776
	Female	50	26		
Age (years)		56.54 ± 10.16	59.59 ± 8.85	2.080	0.039
Smoking (years)	No	101	11	78.069	<0.001
	1–9	24	17		
	10–20	7	22		
	>20	2	16		
Chronic pulmonary disease	No	98	21	31.325	<0.001
	Yes	36	45		
Nodule type	SN	95	18	44.462	<0.001
	mGGN	33	26		
	pGGN	6	22		
Nodule diameter (cm)		4.67 ± 1.24	8.67 ± 2.35	15.768	<0.001
Nodule morphology	Rules	86	29	7.413	0.006
	Irregularities	48	37		
Lobulated	No	108	46	2.967	0.085
	Yes	26	20		
Spiculation	No	112	48	3.256	0.071
	Yes	22	18		
SPN	No	70	40	1.251	0.263
	Yes	64	26		
Tracheal changes	No	121	49	8.941	0.003
	Yes	13	17		
Vascular changes	No	115	48	5.028	0.025
	Yes	19	18		
Average CT (HU)		40.29 ± 8.36	38.49 ± 7.70	1.469	0.144

Note: ① Chronic obstructive pulmonary disease (COPD); ② solid nodule (SN); ③ mixed ground glass nodule (mGGN); ④ pure ground glass nodule (pGGN); ⑤ solitary pulmonary nodule (SPN).

### 3.3. Comparison of lung cancer biomarkers between the two groups

The levels of CEA, NSE, CYFRA21–1, SCCA, and ProGRP in the benign group were all significantly lower than those in the malignant group, with the differences being statistically significant (P < 0.05), as shown in Table 4.

**Table 4.** Comparison of lung cancer biomarkers between the two groups (*x* ± s).

Item	Benign group (n = 134)	Malignant group (n = 66)	*t*	*P*
CEA (ng/mL)	2.81 ± 1.23	7.34 ± 2.26	18.367	<0.001
NSE (ng/mL)	15.67 ± 4.35	28.39 ± 7.16	15.563	<0.001
CYFRA21–1 (ng/mL)	2.08 ± 0.79	3.74 ± 1.35	10.943	<0.001
SCCA (µg/mL)	2.62 ± 0.57	5.73 ± 1.40	22.279	<0.001
ProGRP (pg/mL)	31.50 ± 7.35	75.27 ± 10.36	34.416	<0.001

### 3.4. Multivariate logistic analysis of malignant pulmonary nodules

Logistic regression analysis showed that age, smoking duration over 20 years, chronic lung disease, nodule diameter, irregular nodules, bronchial changes, vascular changes, NSE, CYFRA21–1, and SCCA were all risk factors for malignant pulmonary nodules (P < 0.05), as shown in Table 5.

**Table 5.** Multivariate logistic analysis of malignant pulmonary nodules.

Variable	*β*	*SE*	*Wald x^2^*	*OR*	*P*	95% *CI*
Age	1.041	0.524	3.791	2.832	0.032	1.654–5.578
Smoking >20 years	3.301	0.458	15.737	27.127	<0.001	23.758–32.672
Chronic pulmonary disease	1.541	0.367	11.441	4.670	<0.001	9.615–16.783
pGGN	0.700	0.507	2.723	2.013	0.071	1.236–3.127
Nodule diameter	2.522	0.678	5.486	12.452	0.016	10.479–15.350
Nodule irregularities	1.976	0.480	8.576	7.215	<0.001	6.829–11.435
Tracheal changes	1.792	0.427	9.828	6.002	<0.001	8.769–12.340
Vascular changes	2.117	0.519	7.859	8.310	<0.001	7.560–10.738
CEA	0.666	0.489	2.785	1.947	0.058	1.345–2.770
NSE	1.446	0.403	8.903	4.245	<0.001	3.261–7.249
CYFRA21–1	3.140	0.678	6.831	23.106	0.001	21.098–29.560
SCCA	1.685	0.336	14.925	5.393	<0.001	4.347–10.234
ProGRP	0.162	0.410	0.964	1.176	0.783	0.867–2.430

### 3.5. ROC curve analysis of each indicator for predicting malignant pulmonary nodules

**Figure 1.**
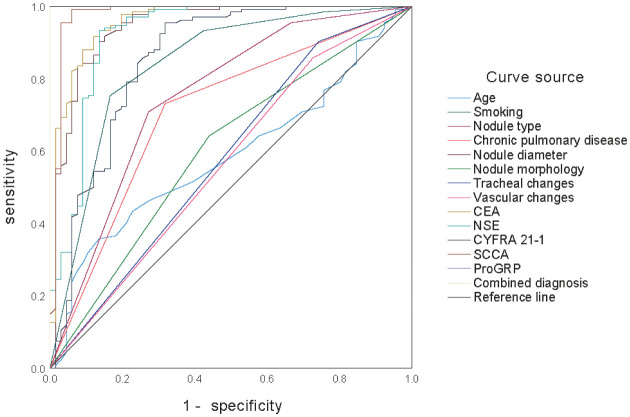
ROC curve analysis of each indicator for predicting malignant pulmonary nodules.

ROC curve analysis showed that age, nodule type, chronic lung disease, nodule morphology, bronchial changes, and vascular changes had moderate predictive value for malignant pulmonary nodules, with AUCs of 0.586, 0.750, 0.707, 0.601, 0.580, and 0.565, respectively. Smoking history, nodule diameter, CEA, NSE, CYFRA21–1, SCCA, and ProGRP demonstrated better predictive value, with AUCs of 0.840, 0.944, 0.958, 0.922, 0.856, 0.978, and 0.990, respectively. The combined diagnosis of all indicators achieved an AUC of 0.993, as shown in Figure 1 and Table 6.

**Table 6.** Sensitivity and specificity of each indicator for predicting malignant pulmonary nodules.

Variable	AUC	Sensitivity (%)	Specificity (%)	Significance	95% *CI*
Age	0.586	35.80	86.40	0.016	0.516–0.655
Smoking	0.840	75.40	83.30	<0.001	0.791–0.889
Nodule type	0.750	70.90	72.70	<0.001	0.691–0.809
Chronic pulmonary disease	0.707	73.10	69.20	<0.001	0.643–0.770
Nodule diameter	0.944	90.30	86.40	<0.001	0.916–0.973
Nodule morphology	0.601	64.20	56.10	0.004	0.533–0.669
Tracheal changes	0.580	90.30	25.80	0.024	0.512–0.649
Vascular changes	0.565	85.80	27.30	0.065	0.497–0.634
CEA	0.958	91.80	87.90	<0.001	0.934–0.981
NSE	0.922	93.30	86.40	<0.001	0.887–0.957
CYFRA21–1	0.856	95.50	68.20	<0.001	0.809–0.903
SCCA	0.978	99.30	93.90	<0.001	0.958–0.998
ProGRP	0.990	99.30	100.00	<0.001	0.975–1.000
Combined diagnosis	0.993	99.30	100.00	<0.001	0.986–1.000

## 4. Discussion

### 4.1. The clinical application value of the CT/AI system for pulmonary nodules

Among the 200 cases of pulmonary nodules analyzed in this study, malignant nodules accounted for 33%, indicating that most pulmonary nodules do not develop into lung cancer. However, because of the significant differences in management strategies between general practitioners and specialists, their assessments of the nature and histological types of pulmonary nodules are often inconsistent [14]. Meanwhile, conventional CT has relatively low sensitivity for determining the nature of pulmonary nodules, and pathological biopsy is an invasive procedure with low efficiency [15]. Compared with traditional methods of pulmonary nodule classification and characterization, the CT/AI system represents a diagnostic approach that integrates efficient and scientific network information technology with radiological imaging. It not only combines multiple imaging features of lesions, shortens diagnostic time, and enables standardized multi-parameter and multi-modality sharing as well as the construction of specific pathological models, but also enhances clinical diagnostic performance and reduces redundant screening and diagnostic harm [16],[17].

Moreover, through techniques such as cluster analysis, image filtering adjustments, reinforcement learning, and decision trees, AI systems can provide precise predictions for CT-based lung cancer diagnosis, pathological markers, surgery, recurrence, and metastasis, significantly improving the image interpretation efficiency of pathologists and radiologists. In addition, employing AI systems as decision-support tools in conjunction with CT can further promote the application of low-dose chest CT, enabling optimal feature, parameter, and model selection for the clinical prevention and management of precancerous lesions, thereby enhancing the early detection rate of lung cancer [18],[19]. However, different studies [20],[21] have indicated that due to insufficient cytological data training and low resolution in distinguishing mixed ground-glass nodules (mGGN), AI-assisted CT evaluation of pulmonary nodules may still misclassify nodule nature. This finding suggests that AI technology, as a general decision-support tool, can only provide a relatively superficial analysis of CT-related parameters of pulmonary nodules. In particular, when patients present with multiple pulmonary diseases, excessive reliance on AI may fail to accurately distinguish overlapping and complex imaging manifestations, potentially misleading clinicians' judgment.

### 4.2. Role and value of lung cancer biomarkers

Carcinoembryonic antigen (CEA) is an important early warning indicator for early-stage cancer and tumor recurrence; in a considerable proportion of post-operative cancer patients whose imaging follow-ups show no abnormalities, significant elevation of CEA usually indicates a higher risk of recurrence [22]. Kuo et al. [23], through cellular experiments and multivariate analysis, demonstrated a significant positive correlation between CEA expression and tumor metastasis, and found that CEA helps guide adjustments to radiotherapy and chemotherapy regimens and monitor prognosis. A related meta-analysis [24] also showed that CEA and CYFRA21–1 have strong practicality and objectivity in assessing treatment efficacy and tumor staging response. This is because CEA serves as a specific targeting system in radiotherapy, capable of reflecting tumor cell activity in real time (including cell morphology, size, and proliferation rate), thereby avoiding insufficient or excessive radiation doses that may affect clinical efficacy [25]. Therefore, the sensitivity, specificity, accuracy, misdiagnosis rate, and missed diagnosis rate of the CT/AI system combined with lung cancer biomarkers were all significantly better than those of the CT/AI system alone.

In addition, Rumende et al. [26] studied the one-year survival rate of patients with advanced non-small cell lung cancer (NSCLC) and found that higher CYFRA21–1 levels were associated with shorter survival times and poorer chemotherapy tolerance. Therefore, CYFRA21–1 has considerable predictive potential for tumor progression and remission. Neuron-specific enolase (NSE) is a specific marker of neuroendocrine cells, with a positive diagnostic rate of up to 90% in NSCLC. NSE not only effectively differentiates NSCLC from small cell lung cancer (SCLC) but also assesses the impact of chemotherapy on the neuroendocrine system of patients [27]. These findings indicate that CEA, CYFRA21–1, and NSE have strong predictive potential for tumor progression and remission, and that lung cancer biomarker detection can effectively compensate for the limitations of the CT/AI system.

In the study by Trulson et al. [28], the combined diagnostic AUC for NSE, CEA, and CYFRA21–1 was 0.93; for ProGRP and NSE, it was 0.89; and for NSE and CEA, it was 0.86. In this study, the combined AUC of all indicators was 0.993, with sensitivity and specificity both exceeding 90%, and individual predictive values of CEA, NSE, CYFRA21–1, SCCA, and ProGRP all greater than 0.80. These findings suggest that combining CT/AI systems with lung cancer biomarkers offers strong flexibility and feasibility for diagnosing pulmonary nodules, allowing for various combination strategies based on clinical needs. For example, Hou et al. [29] extracted imaging features of lesions using the CT/AI system and combined them with patients' clinical characteristics and markers such as CEA and CYFRA21–1 to diagnose pulmonary nodules, significantly improving the predictive probability of malignancy and enhancing the accuracy of surgical decision-making.

### 4.3. Value and limitations of the CT/AI system combined with lung cancer biomarkers

Typically, malignant nodules exhibit significantly faster growth rates, larger diameters or average volume on CT, and more pronounced bronchial and vascular changes compared to benign nodules. Some patients may also present with symptoms such as cough with sputum, fever, and chest pain [30]. However, ROC curve analysis indicated that age, nodule type, chronic lung disease, nodule morphology, bronchial changes, and vascular changes have only moderate predictive value for malignant pulmonary nodules, which is related to the heterogeneity and variability of pulmonary nodules. Additionally, cases with rapid growth or new nodules detected during follow-up are not always malignant [31]. For example, pulmonary fibromas, hamartomas, and inflammatory nodules, although sometimes larger in size and associated with elevated lung cancer biomarkers, are benign and have favorable prognoses [32]. Conversely, nodules smaller than 5 mm exhibiting pleural retraction, spiculation, or lobulation signs—especially with deep lobulation—have a high probability of malignancy and poorer prognosis [33]. Moreover, AI analysis of the dynamic changes in biomarkers such as CYFRA21–1, CEA, and NSE before, during, and after treatment, as well as during follow-up, can help establish survival models and databases for different lung cancer types, providing scientific evidence for intervention strategies at various stages of pulmonary nodule development [34]. Therefore, diagnosing the nature of pulmonary nodules through the combined use of CT/AI systems and lung cancer biomarkers is of great necessity.

A multicenter retrospective study [35] confirmed that dynamic monitoring of serum tumor markers in lung cancer patients not only improves the treatment efficacy for squamous cell carcinoma and adenocarcinoma but also enhances patients' quality of life and reduces treatment costs. Additionally, the CT/AI system can effectively identify the type of pulmonary nodules and predict the occurrence and progression of other chronic lung diseases, thereby supporting public health improvement [36]. It should be noted, however, that several limitations remain in the combined detection using CT/AI systems and lung cancer biomarkers: (1) most patients detected with pulmonary nodules do not adhere to standardized and complete CT/AI or biomarker follow-up plans before the disease progresses to lung cancer [37],[38]; (2) for patients undergoing immunotherapy or targeted therapy, changes in ProGRP, CEA, NSE, SCCA, and CYFRA21–1 are influenced by multiple factors, which may interfere with test results and increase false-positive rates; (3) although AI technology offers significant advantages in identifying typical CT features of pulmonary nodules and in summarizing and extracting relevant parameters, the absence of unified standards limits its ability to perform deep understanding and comprehensive judgment of complex cases [39]; and (4) the generalizability of CT/AI system models is insufficient [40],[41], as variations in CT equipment, scanning parameters, individual differences, and serum testing methods can all impact the accuracy of CT/AI combined biomarker diagnosis.

## 5. Conclusions

**Figure 2.**
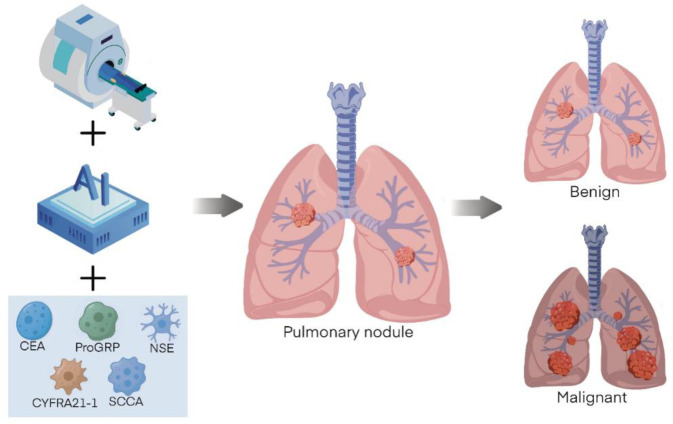
CT/AI systems and lung cancer biomarkers demonstrate high predictive value for determining the nature of pulmonary nodules.

The combined use of CT/AI systems and lung cancer biomarkers demonstrates high predictive value for determining the nature of pulmonary nodules (Figure 2), and the occurrence of malignant nodules is mainly associated with risk factors such as age, smoking, and chronic lung disease. Clinically, targeted interventions can be implemented based on these factors to control the development and progression of lung cancer. However, AI technology currently serves only as an auxiliary decision-support tool and still has notable limitations in clinical applications. To achieve widespread, accurate, real-time, and continuous prediction and targeted treatment of malignant pulmonary nodules, it is necessary to continuously optimize CT/AI systems and strengthen AI dataset training while ensuring patient privacy, thereby further improving the flexibility, simplicity, and accuracy of combined diagnostic approaches.
